# Engineering a Fungal Non-Reducing Polyketide Synthase with an Apparently Inactive Product-Template Domain Reveals Insights into the Catalytic Reprogramming

**DOI:** 10.3390/ijms27125534

**Published:** 2026-06-18

**Authors:** Ruya Yin, Yifei Qin, Xingrui Liang, Ziqi Zhai, Mengwei Zhang, Dan Xu, Ligang Zhou, Daowan Lai

**Affiliations:** State Key Laboratory of Agricultural and Forestry Biosecurity, MARA Key Lab of Pest Monitoring and Green Management, College of Plant Protection, China Agricultural University, Beijing 100193, China; ruyayin@cau.edu.cn (R.Y.); s20253193760@cau.edu.cn (Y.Q.); xingruiliang@cau.edu.cn (X.L.); ziqizhai@cau.edu.cn (Z.Z.); mengweizhang@cau.edu.cn (M.Z.); cauxudan@cau.edu.cn (D.X.); lgzhou@cau.edu.cn (L.Z.)

**Keywords:** polyketide synthase, product template, C-methyltransferase, domain engineering, catalytic reprogramming, polyketide, sorbicillin, synthetic biology, biosynthesis

## Abstract

Fungal iterative non-reducing polyketide synthases (NR-PKS) contain a unique product template (PT) domain for aromatic cyclization. Among them, some NR-PKSs, such as the sorbicillin NR-PKS (SorB), have an apparently inactive PT. It is unknown what role such PT plays in NR-PKS programming. In this study, the PT domain of SorB was first dissected and engineered. Removal of the PT domain from SorB did not change the product profile, but the yield decreased. Meanwhile, a significantly lower transcriptional level of the ketoacyl synthase (KS) domain was observed in the knockout mutant (*UvSorB∆PT*). Phylogenetic tree analysis and multiple sequence alignments revealed this PT belongs to group I (C2–C7, monocyclic ring), and mutations were found at catalytic dyad sites when compared with functional fungal PTs. However, mutating these residues back to the conserved ones did not give rise to products corresponding to a functional PT, but rendered the NR-PKS unproductive. Likewise, removal of the C-methyltransferase (CMT) domain from SorB destroyed the polyketide production. Furthermore, in an attempt to alter the methylation pattern, mutations of the key substrate-binding sites of the CMT domain were made. Site-directed mutations of the C-MT led to cessation of the polyketide production. This reveals CMT is vulnerable to engineering in a collaborating NR-PKS (SorB). These results provide additional insights for catalytic reprogramming in fungal NR-PKS.

## 1. Introduction

Fungal polyketides represent a unique class of secondary metabolites featuring complex structural diversity and extensive biological functions, which span medicinal active substances to harmful mycotoxin compounds. The biosynthesis of these compounds is predominantly catalyzed by iterative type I polyketide synthases (PKSs), including non-reducing (NR), partially reducing (PR) and highly reducing (HR) PKSs [1]. NR-PKSs often contain starter-unit acyltransferase (SAT), β-ketoacyl synthase (KS), acyltransferase (AT), product template (PT), acyl carrier protein (ACP), and product release domains, along with an optional C-methyltransferase (CMT) domain, but lack β-carbonyl modification modules to facilitate the formation of nonreduced products, including poly-β-ketone and aromatic metabolites. Notably, the SAT and PT domains are specifically conserved in fungal NR-PKSs. The SAT domain initiates polyketide chain assembly by catalyzing the transfer of specific starter substrate to the ACP domain [2]. The KS domain is responsible for elongating the starter unit to a designated chain length. Subsequently, the PT domain mediates specific aldol cyclization and aromatization reactions [3]. Finally, products are released with the assistance of thioesterase (TE), reductase (R), or other releasing domains [3,4].

The PT domain is unique in NR-PKSs and responsible for the formation of aromatic rings. As for fungal NR-PKS products, three different first ring cyclization patterns (C4-C9, C2-C7, and C6-C11) have been reported [3]. Among which, the monocyclic aromatic polyketides were mostly from the C2-C7 cyclization pattern; however, some were generated by NR-PKS with a seemingly inactive PT domain, such as SorB, ClaF, and MPAS, which produce sorbicillin (SorB) and clavatol (ClaF) via Knoevenagel cyclisation, or 3-methylphloracetophenone (MPAS) via Claisen/Dieckmann condensation by the TE/Claisen cyclase (TE/CLC) domain. From these nascent polyketides, a diverse array of complex structures are produced, such as sorbicillinoids [5,6], penilactones [7], and usnic acid [8]. It is unclear what role such PTs play in NR-PKSs.

**Figure 1:**
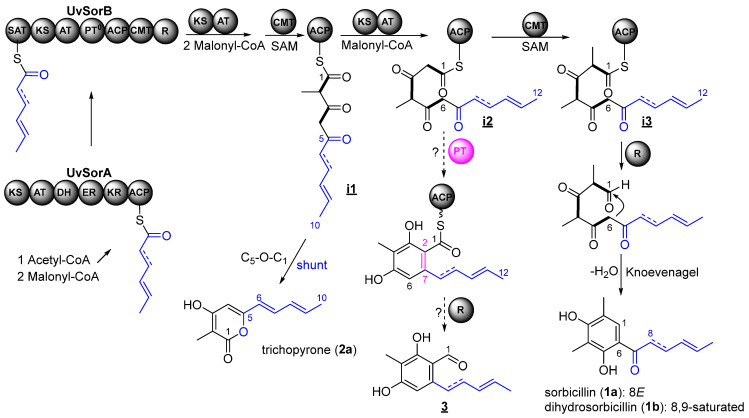
Biosynthetic pathway of sorbicillin and related metabolites. Note: The proposed intermediates were underlined and in bold, while the experimentally confirmed products are in bold. Hypothetical branching events were indicated with dashed arrows. The blue colored fragment in each structure is from the starter unit biosynthesized by the HR-PKS UvSorA, while the PT domain catalyzed cyclization is shown in purple. SAT: starter-unit acyltransferase; KS: β-ketoacyl synthase; AT: acyltransferase; PT: product template; ACP: acyl carrier protein; CMT: C-methyltransferase; R: reductase; DH: dehydratase; ER: enoyl reductase; KR: ketoreductase.

To address this issue, we turned to the sorbicillin NR-PKS SorB, which accepts a sorboyl side chain provided by the collaborating HR-PKS (SorA) [9,10,11]. SorB contains SAT/KS/AT/PT/ACP/CMT/R domains, and elongates the sorboyl starter three times with malonyl CoA by the catalysis of KS and AT domains to generate the hexaketide skeleton (Figure 1). During the elongation, α-methylation occurs at C2 and C4 by the catalysis of CMT, before a reductive release with the R domain to afford an aldehyde that was spontaneously cyclized to give the final product (dihydro)sorbicillin (**1a**/**1b**) (Figure 1). Obviously, the PT domain seems inactive judging from the proposed biosynthetic pathway (Figure 1).

In this study, we engineered the PT and CMT domain of UvSorB with various efforts. The results are reported herein.

## 2. Results

### 2.1. Engineering the PT Domain of UvSorB

#### 2.1.1. Phylogenetic Tree and Sequence Comparison of PT

**Figure 2:**
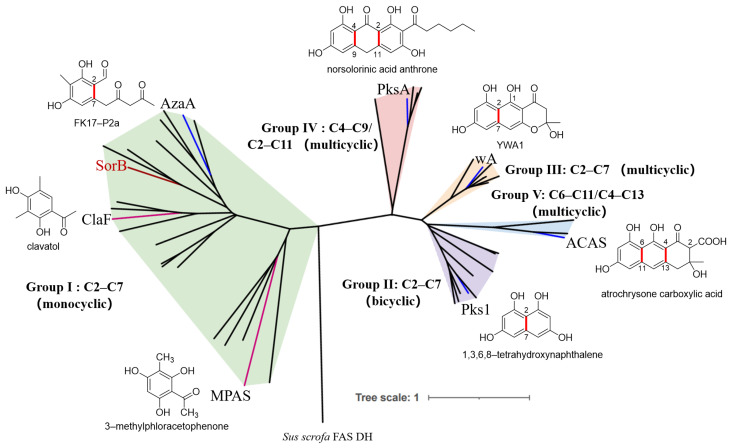
Phylogenetic analysis of the UvSorB PT domain with group I-V PT domains. *Sus scrofa* FAS DH domain was selected as an outgroup. Bootstrap support values were calculated with 1000 replicates. The representative PT in each group was indicated with a blue line, with its product shown nearby. The UvSorB_PT domain is indicated with a red line. The ClaF_PT and MPAS_PT are shown in purple, which seem non-functional judging from the structure of clavatol and 3-methylphloracetophenone. A more comprehensive tree is shown in Figure S1.

In the literature, fungal PTs could be classified into five groups according to their products that differed in cyclized position and the number of rings formed [12]. Similarly, we constructed a phylogenetic tree for the PT domain of UvSorB. As shown in Figure 2, UvSorB_PT belongs to the clade (group I) that catalyzes the C_2_-C_7_ cyclisation to form a monocyclic product. Interestingly, the PT of ClaF was also observed in this same clade. The product of ClaF is clavatol [7], which is an analogue of sorbicillin, by having an acetyl side chain instead of a sorboyl one (Figure 2). By analogizing to the biosynthetic pathway of sorbicillin, ClaF_PT should be non-functional as well. Interestingly, MPAS_PT was also in the same group, but in the other subgroup (Figure 2). Apparently, this PT is also not active, as deduced from the product structure, i.e., 2,4,6-trihydroxy-3-methyl-acetophenone (also known as 3-methylphloracetophenone), in which the C-C connection between the acetyl-linked C_1_ and the hydroxyl-bearing C_2_ is formed via Claisen condensation by the TE/CLC domain.

**Figure 3:**
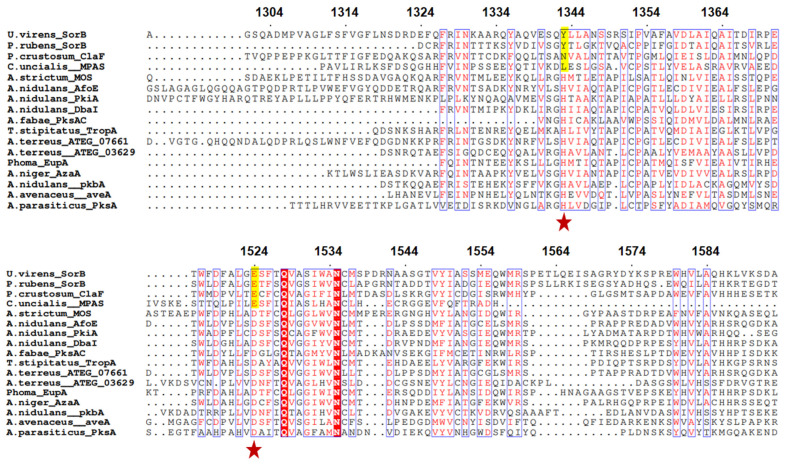
Multiple sequence alignment (partial) of the UvSorB_PT domain with other PT domains in group I (except PksA). Catalytic dyad residues were marked with a red star. The residues with high similarity were shown in red, while those highlighted in red background were conserved among all the sequences. Similar residues were boxed for a better comparison. For full sequence alignment, see Figure S2 in the Supplementary File.

A previous study suggested that the catalytic mechanism for PT is conserved in fungal NR-PKSs [3]. Based on the solved PT structure and mutagenesis of key residues, it has been confirmed that the His1345 in PksA served as the catalytic base to deprotonate the α-carbon of the 1,3-dicarbonyl intermediate to form an enolate which attacks the keto group locating at five carbons away to generate the cyclic product *via* aldo cyclization. The Asp1543 stabilizes His1345 by hydrogen bonding. Mutation of both residues to Ala in PksA resulted in no detectable activity [3]. Next, sequence alignment of the UvSorB_PT domain with homologues in the same group reveals that this PT lacks a classic catalytic dyad (His1345/Asp1543) as represented by PksA [3]; instead, a Tyr1343 and Glu1524 residue was found in the corresponding site, respectively (Figure 3). Similarly, a mutation from His to Asn, and from Asp to Glu, was observed in the corresponding sites of ClaF_PT, while His to Leu and Asp to Glu was found in MPAS_PT. All of this information suggests that the UvSorB_PT domain (and perhaps its analogue ClaF_PT, MPAS_PT) is likely inactive, which was confirmed by the following domain-deletion experiment (*vide infra*).

#### 2.1.2. The PT Domain Is Nonfunctional in UvSorB

Judging from the biosynthetic pathway of (dihydro)sorbicillin (Figure 1), and also the bioinformatic analysis, the PT domain seems to be not required for the biosynthesis of sorbicillin. To test this hypothesis, the PT domain was removed from UvSorB. Then, the truncated NR-PKS (*UvSorB∆PT*) was co-expressed with *UvSorA* in *Aspergillus oryzae* NSAR1, which is a robust heterologous host for investigating the biosynthesis of fungal metabolites [13,14]. Three independent transformants were selected for further cultivation. Finally, the crude extracts from fungal cultures were analyzed via LC-MS.

**Figure 4:**
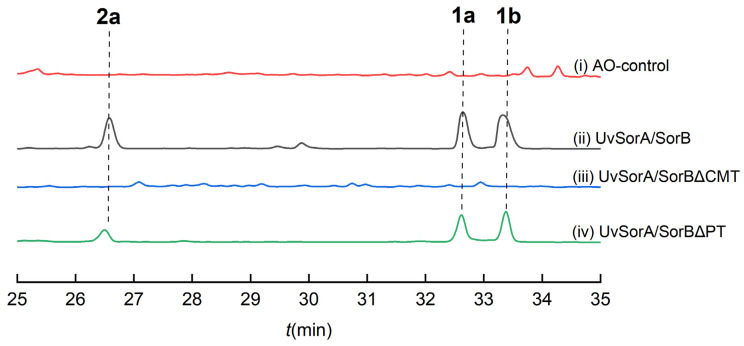
HPLC analysis of the co-expression of *UvSorA* and the truncated *UvSorB* in *A. oryzae* (λ = 350 nm). (**i**) Empty vector control, (**ii**) full *UvSorB*, and *UvSorB* without (**iii**) CMT, or (**iv**) PT domain. For chemical structures, see Figure 1.

As shown in Figure 4, the NR-PKS without PT domain still produced sorbicillin (**1a**) (Figure S4) and dihydrosorbicillin (**1b**) (Figure S5) as the major products, and a pyrone shunt, named trichopyrone (**2a**), (Figure S6), when co-expressed with *UvSorA* in *A. oryzae* (Figure 4, trace iv). These metabolites were also detected in the strain co-expressing *UvSorA* and *UvSorB* (Figure 4, trace ii) but with higher abundance. Comparing the peak areas revealed that the yield of **1a** and **1b** with a complete sequence of *UvSorB* (trace ii) was 3.5 and 6.2 times as high as that of the *∆PT* mutant (trace iv), respectively.

Next, we compared the transcriptional level of the KS domain for the knockout mutant (*UvSorB∆PT*) with the wild type (WT, *UvSorB*) using qRT-PCR. It was found that the relative expression level in WT was 24.5 times as high as that of the mutant. This might partly explain the higher product yields in the WT.

#### 2.1.3. Revert the Catalytic Residues to the Evolutionarily Conserved Ones

As stated above, the mutations at the catalytic dyad residues render the UvSorB_PT inactive. We envisioned that if we back-mutated these two residues to the conserved amino acids by site-directed mutagenesis, the function of the PT might be restored.

**Figure 5:**
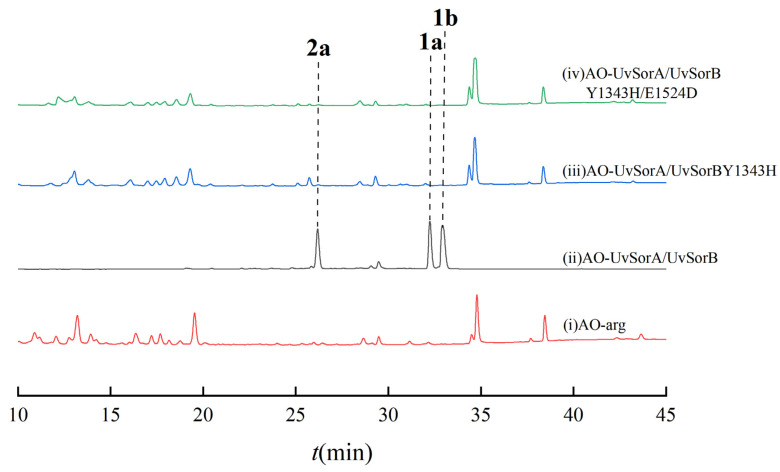
HPLC analysis of the co-expression of *UvSorA* and the *UvSorB PT* mutants in *A. oryzae* (λ = 210 nm). (**i**) Empty vector control; (**ii**) *UvSorB*; (**iii**) singular mutant Y1343H, and (**iv**) double mutant Y1343H/E1524D of *UvSorB*.

For this purpose, a single mutation of Y1343H and a double mutation of Y1343H/E1524D were constructed by overlap PCR and confirmed by Sanger sequencing. The PCR product containing the desired mutation was digested and ligated into the expression vector, which was co-expressed with *UvSorA* in *A. oryzae*, to give the corresponding mutants. All the mutants were confirmed to contain the desired mutation, and then grown in DPY media, together with the wild type and empty vector control. After 7 days, the culture was harvested and analyzed by HPLC (Figure 5).

It was expected that a functional PT of group I would catalyze a C2-C7 cyclisation on the thioester intermediate **i2** to give a monomethylated benzothioate, which could be reductively released to afford a monomethylated benzaldehyde (**3**) (Figure 1). However, unlike this expectation, neither the single (Figure 5, iii) nor the double mutation (Figure 5, iv) of the PT domain produced any new polyketide. On the contrary, the production of sorbicillin and its analogues (**1a**/**1b**, **2a**) was not detectable in these mutants via LC-MS analysis. These results indicated that the restoration of these residues confers a dysfunctional PKS in vivo.

It is likely that the CMT is competing with PT to work on the thioester-intermediate (**i2**) when the polyketide chain ceases extension (Figure 1). If C_2_ is methylated by CMT, the acidity of the proton at C_2_ decreases. Even if the methylated C2 is able to deprotonate and attack the C7 keto, the subsequent dehydration is impossible from the resulting aldol (no proton at C2 anymore); hence, the C_2_-C_7_ cyclisation cannot happen. On the contrary, the PT domain could catalyze the nucleophilic attack from C_2_ to C_7_ and the subsequent dehydration, when C_2_ is not methylated (Figure 1). It is still unknown why the restored PT does not outperform the CMT, and why these mutations in PT render the PKS dysfunctional.

### 2.2. Engineering the CMT Domain of UvSorB

#### 2.2.1. CMT Is Indispensable for the PKS Function

As mentioned above, the CMT domain might have an important role in the normal function of UvSorB. Then, we removed the CMT domain from *UvSorB*, and co-expressed the truncated NR-PKS (*UvSorB∆CMT*) with *UvSorA* in *A. oryzae*, which led to cessation of the production of sorbicillin (**1a**) and dihydrosorbicillin (**1b**), as expected (Figure 4, iii). Surprisingly, no pyrone shunt could be detected in the culture, whereas pyrones are commonly seen in in vitro domain reconstruction of fungal NR-PKSs regardless of the presence of CMT [15,16].

#### 2.2.2. Multiple Sequence Alignment Revealed Residues Correlated to Methylation Pattern

It is known that chain elongation by KS and C-methylation by CMT are competing processes, and the methylation pattern is determined intrinsically by the sequence of CMT to a larger extent [16]. Using the CMT domain of citrinin PKS (PksCT) as a template [15], which has a solved protein structure, sequence alignment was conducted between homologues with known products [17].

Since CMT catalyzes methylation after polyketide extension (at α-position), we curated the methylation pattern according to the methylating events following extension(s) with malonyl-CoA regardless of a simple or complex starter. For example, methylation occurs following both the 2nd and 3rd round of extension in SorB (referred to as pattern #2,3). Similarly, methylation occurs after the 2nd or 3rd round of extension was designated as pattern #2 and #3, respectively.

**Figure 6:**
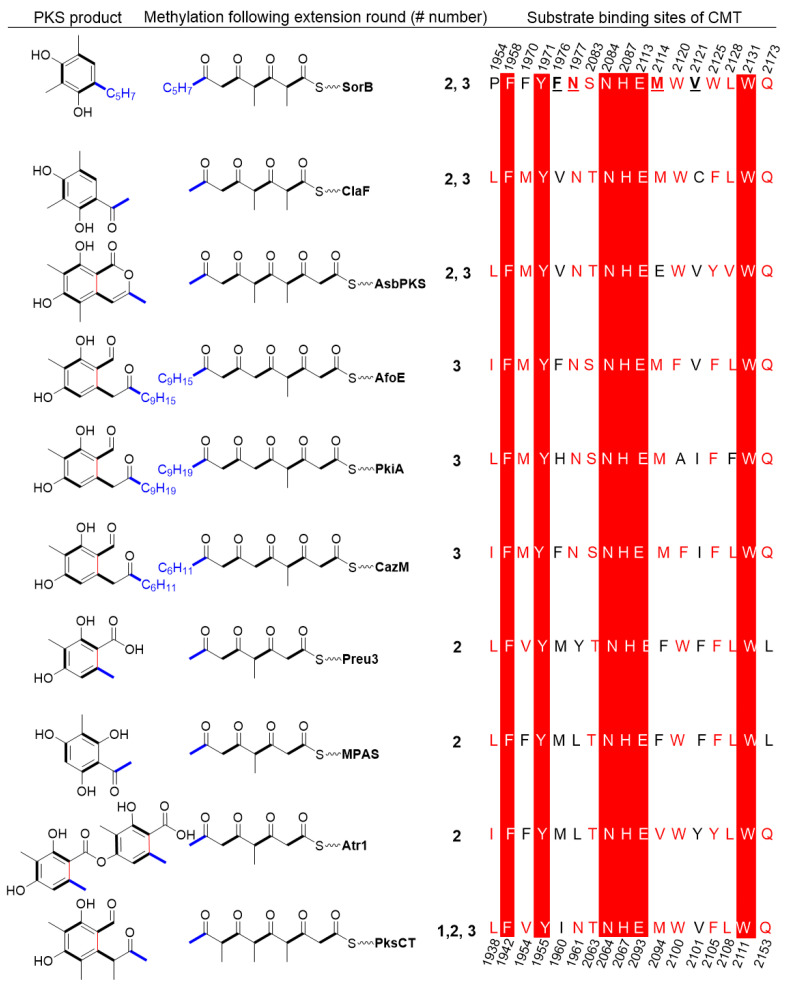
Multiple sequence alignment of the substrate-binding sites of CMTs (**right**), with their corresponding methylation pattern (**middle**) and PKS products (**left**) shown. Note: the starter unit in blue, the extension unit (from malonyl CoA) in bold and black, and the PT-catalyzed cyclization in red. The amino acid residues with high similarity were shown in red, while those highlighted in red background were conserved among all the sequences. The residues selected for site-directed mutagenesis in UvSorB are underlined.

As shown in Figure 6, apart from the highly conserved catalytic dyad His2087/Glu2113 and the strictly invariant residues Phe1958, Tyr1971, Asn2084 and Trp2131, residues at positions 1976, 1977, 2114 and 2121 (numbered according to UvSorB) exhibit correlations with the methylation patterns. Specifically, the 1976 site is uniformly methionine in pattern #2. In this pattern, the predominant amino acids at positions 1977, 2114 and 2121 are leucine, phenylalanine and phenylalanine, respectively. By contrast, the amino acids in pattern #3 share more similarity with pattern #2,3 at these variable sites.

#### 2.2.3. Attempt to Alter the Methylation Pattern Through Site-Directed Mutation of Key Residues

It is intriguing to see whether the methylation pattern could be engineered by mutation of key substrate-binding residues of CMT. As depicted in Figure 1, if the methylation pattern of UvSorB_CMT was altered from pattern #2,3 to pattern #2, the intermediate **i3** might not be formed. If this were succeeded, restoration of PT on such a mutation would likely generate **3** *via* intermediate **i2**.

As stated above, residues at 1976, 1977, 2114 and 2121 (Figure 6) seem to correlate with the methylation pattern. For residue at 1976, methine is exclusively seen in pattern #2; at 1977, Leucine is seen at a higher frequency (2/3) than tyrosine (1/3); at 2114, phenylalanine is seen at a higher frequency (2/3) than valine (1/3); while at 2121, phenylalanine is seen at a higher frequency (2/3) than tyrosine (1/3). Then, site-directed mutations of the Phe1976 residue in UvSorB to Met, Asn1977 to Leu, Met2114 to Phe, and Val2121 to Phe were achieved and confirmed by sequencing. The mutated *UvSorB* was co-expressed with *UvSorA* in *A. oryzae*, respectively, to give four single mutants (F1976M, N1977L, M2114F, V2121F) and a quadruple mutant (F1976M/N1977L/M2114F/V2121F). After fermentation and extraction, the secondary metabolic profiles of these mutants were analyzed by LCMS.

**Figure 7:**
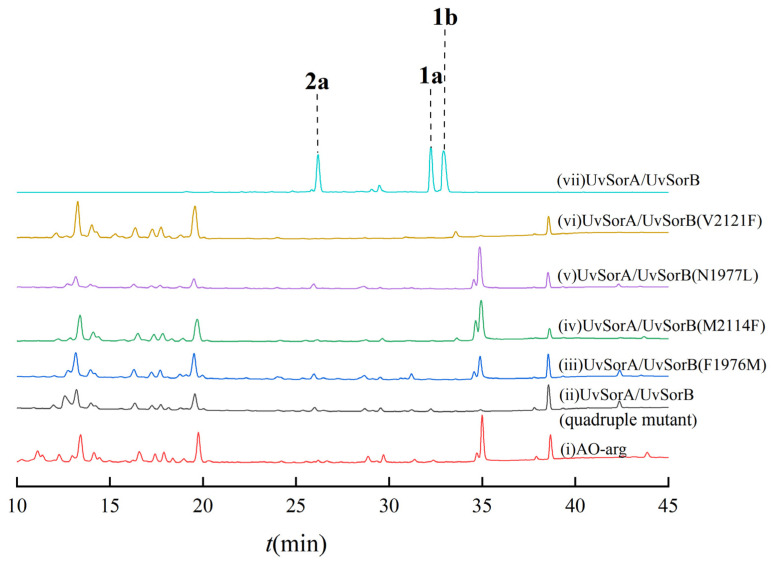
HPLC analysis of the extract from co-expression of *UvSorA* and various CMT mutants of *UvSorB* in *A. oryzae* (210 nm). (**i**) Empty vector control; (**ii**) quadruple mutation, and (**iii**–**vi**) single mutation of *UvSorB* CMT; (**vii**) intact *UvSorB*.

As a result, the AO-UvSorA/SorB transformants produced sorbicillin (**1a**) and dihydrosorbicillin (**1b**), and the empty vector control (AO-control) did not, as expected (Figure 7, i and vii). Five clones of AO-UvSorB-F1976M, -N1977L, -M2114F and -V2121F were examined, but none of these produced (dihydro)sorbicillin (Figure 7, iii–vi). Similarly, five clones containing the quadruple mutations were isolated but also did not produce (dihydro)sorbicillin (Figure 7, ii). Fermentation was repeated two times for all the mutants, and the initial observations were confirmed by LC-MS analyses.

Meanwhile, we compared the LC-MS chromatogram of these mutants with AO-control to look for new polyketides resulting from a possible change in the intrinsic methylation programming of UvSorB. Specific ions corresponding to possible demethylated analogues of (dihydro)sorbicillin and its pyrone shunts were monitored with extracted ion chromatography (EIC), with mass tolerance set at ±0.05 Da. However, no convincing products could be detected, though a deprotonated molecular peak at *m*/*z* 193.08 (referred to as **2b**) could be observed in all mutants (ESI-MS negative mode), while absent in negative (empty vector) and positive control (UvSorA/UvSorB) (Figure S3). The HRMS analysis revealed its molecular formula as C_11_H_14_O_3_ (Figure S7), which happened to contain two more hydrogen atoms than that of the pyrone shunt, trichopyrone (**2a**). However, this compound (**2b**) cannot be isolated for NMR analysis due to its lower abundance. Taken together, these results revealed that all these residues are vital to the function of CMT.

#### 2.2.4. qRT-PCR Analysis of the CMT-Mutants

**Figure 8:**
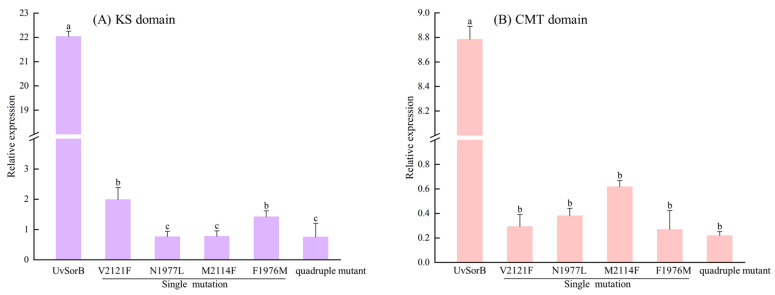
qRT-PCR analysis of the CMT mutants. (**A**) Relative expression level of the KS domain. (**B**) Relative expression level of the CMT domain. Different letters indicate significant difference (*p* < 0.05), while identical letters suggest no significant difference.

Next, we checked the transcriptional level of each CMT mutant using qRT-PCR. Here, we probed two important domains: the KS domain, the central part of PKS, which catalyzes polyketide extension, along with the CMT domain (for C-methylation). As shown in Figure 8, all the mutants displayed significantly lower expression levels than the wild type with regard to both KS and CMT domains. As for the transcriptional levels of the KS domain, the CMT mutants were 11.1~29.3 times lower than that of the WT (*UvSorB*) (Figure 8A). Among them, the quadruple mutant and the single mutant of N1977L and M2114F displayed similar low levels, while the remaining two single mutants of V2121F and F1976M showed relatively higher levels. On the other side, the CMT mutants were 14.2~40.0 times lower than the WT, regarding the transcriptional levels of the CMT domain (Figure 8B); however, there was no significant difference among these mutants. In both cases, the lowest level was recorded for the quadruple mutant.

## 3. Discussion

Although PT domain appears to be specifically present in NR-PKS, its function has not been revealed where the PT domain seems non-functional. In this study, the sorbicillin NR-PKS UvSorB which has an apparently inactive PT domain was investigated. Through phylogenetic analysis and multiple sequence comparison, the conserved catalytic dyad (H1345/D1543) in functional PT (PksA) was found to mutate to Y/E, N/E, and L/E in SorB, ClaF, and MPAS, respectively (Figure 3). Then, back-mutation of both catalytic residues in UvSorB to the revolutionarily conserved residues was carried out. However, no products corresponding to a functional PT could be obtained (Figure 5). Instead, such mutations cause the NR-PKS to stop functioning. In contrast, removal of the PT domain does not affect the production of sorbicillins albeit with a relative lower yield (Figure 4, trace iv). Consistently, transcriptional analysis of the KS domain revealed that the knockout mutant has a much lower transcriptional level than the WT. These results suggest that PT contributes to a high catalytic efficiency, plausibly by affecting the protein integrity, or domain–domain interaction, though not necessitated for the catalysis, which requires further study.

The competition between CMT and KS in fungal NR-PKS for acyl-ACP intermediate has been proposed for exploring polyketide C-methylation [16]. Similarly, the engineered PT and CMT might compete for the thioester intermediate (**i2**) for further programming (Figure 1); it was unknown why the restored PT did not outcompete the CMT, and why these mutations in PT render the PKS not productive. There could be many explanations, such as impaired protein folding, altered conformational dynamics, defective ACP–domain interactions, disrupted substrate channeling, or reduced protein stability, which remains to be investigated using substrate mimic of **i2** and dissected protein in vitro.

This pinpoints the importance of the CMT domain in sorbicillin polyketide biosynthesis in vivo, which was supported by the domain deletion experiment, in which the polyketide production was shut down when the CMT domain was removed (Figure 4, trace iii).

From the multiple sequence analysis, the methylation pattern seems well correlated to the CMT binding residues regardless of the starter unit (Figure 6). As for NR-PKSs with an acetyl-loading SAT, mutation of the substrate binding sites of CMT has led to products with an altered methylation pattern in an earlier study, though with less efficacy [17]. It was found that a single mutation (F2044L) of a 3,5-dimethylorsellinic acid synthase (NvfA) led to production of 5-methylorsellinic acid, albeit minor, in *A. oryzae* NSAR1; E2052M/L mutants of AsbPKS produced a trace compound with one additional methyl group as detected by LC-MS [17]. However, it is unknown if the methylation pattern could be altered in a collaborating NR-PKS prior to this study.

In the current study, we engineer the CMT of a collaborating NR-PKS (UvSorB), aiming at remodeling the methylating events (from pattern #2,3 to #2). Single mutations at four different sites and quadruple mutations of UvSorB_CMT have been created successfully. However, no sorbicillin analogues could be detected from the CMT mutants (Figure 7), and neither were any demethylated products of this kind found. Further analysis of the transcriptional levels of these CMT mutants (Figure 8) revealed they displayed a much lower level than the WT regarding the KS and CMT domains. This suggested that the mutations rendered the PKS less efficient in chain extension and methylation. These results implied that those residues are important for the CMT function.

It is unclear whether the point mutations to the CMT domain of UvSorB had the desired effect. If it succeeded, the intermediate **i2** would be accumulated (Figure 1), which was not a native substrate for the R domain, however. This might cause the hexaketide to release as a polyketo-acid, which could be subjected to degradation via β-oxidation in vivo, and this requires further investigation. These results somehow mirrored those of Cox and co-workers, who attempted to alter the methylation pattern by mutating the CMT domain of squalestatin tetraketide synthase (a HR-PKS) [18], which led to no effect or total abrogation of polyketide production. They proposed that the product might have been formed but transformed in vivo, likely through β-oxidation, as the proposed oct-2-enoic acid could not be detected after feeding it to *A. oryzae* for 24 h. From this aspect, it is possible that the methylation pattern might have changed from #2,3 to #2 after point mutation, resulting in a pre-matured release of the polyketide as pyrone shunt(s); however, more evidence should be obtained. One limitation for in vivo experiments is the presence of other enzymes, which might cause chemical transformation of the target products. Without analysis of purified enzymes, it remains difficult to distinguish catalytic effects from indirect consequences on protein assembly or stability. For future investigations, in vitro studies on the effect of dissected domains using specific substrates would be required.

## 4. Materials and Methods

### 4.1. Reagents and Chemicals

All solvents and reagents used in the experiments were purchased from Sigma-Aldrich (St. Louis, MO, USA) and Fisher Scientific (Waltham, MA, USA). Solvents for liquid chromatography–mass spectrometry (LC-MS) and high performance liquid chromatography (HPLC) analysis were of chromatographic grade. Conventional molecular biology procedures were performed in accordance with standard protocols, and relevant molecular biology kits were used following the manufacturers’ instructions. DNA polymerases for PCR amplification and restriction endonucleases were obtained from Takara Bio Inc. (Otsu, Shiga, Japan).

### 4.2. Liquid Chromatography and LC-MS Analysis

HPLC-DAD analysis was performed using a Shimadzu LC-20A system (Shimadzu Corporation, Kyoto, Japan) equipped with an SPD-M20A photodiode array detector and a Phenomenex (Torrance, CA, USA) C18 chromatographic column (250 mm × 4.6 mm, 5 μm) Methanol was used as solvent A, and water containing 0.2% trifluoroacetic acid was used as solvent B. The sample analysis procedure was as follows: gradient elution with 10% to 100% solvent A was applied within 0–33 min; subsequently, the column was rinsed with 100% solvent A from 33 to 45 min; finally, the column was re-equilibrated with 10% solvent A during 45–56 min. The flow rate was set at 0.85 mL/min, and the detection wavelength was 210 nm.

LC-DAD-MS data were acquired using an Agilent 1260 HPLC system coupled with a Q-TOF 6520 mass spectrometer (Agilent Technologies, Santa Clara, CA, USA), equipped with a Phenomenex C18 column (150 mm × 2.0 mm, 3 μm). The mass spectrometer was operated in both ESI^+^ and ESI^−^ modes with a mass range of 50–1400 *m*/*z*. The mobile phases consisted of solvent A (water containing 0.1% formic acid) and solvent B (acetonitrile containing 0.1% formic acid). The flow rate was set to 0.25 mL/min. The elution procedure was as follows: gradient elution with 10% to 90% solvent B within 0–18 min, followed by column rinsing with 90% solvent B from 18 to 23 min, and column re-equilibration with 10% solvent B during 23–30 min. Data analysis was performed using MassHunter B.07.00 software.

Extracted ion chromatography (EIC) was applied to monitor specific ions corresponding to (dihydro)sorbicillin and its potential demethylated analogs, with the mass tolerance set at ±0.05 Da. Sorbicillin (**1a**) (molecular mass 232.1) and dihydrosorbicillin (**1b**) (molecular mass 234.1) were monitored under both negative and positive mode of ESI, while the pseudo-molecular ions for potential demethylated analogs were searched at *m*/*z* 217.1, 219.1 [M−H]^−^ in the negative mode, and at *m*/*z* 219.1, 221.1 [M+H]^+^; 241.1, 243.1 [M+Na]^+^ in the positive mode, respectively. The product should appear only in the mutants but not in the control. Similarly, special ions corresponding to the hypothesized pyrone shunts of the proposed pathway were searched.

### 4.3. HRMS

High-Resolution Mass Spectrometry (HRMS) data were determined using an Agilent 6500 Q-TOF mass spectrometer coupled with an HPLC system (Agilent Technologies, Santa Clara, CA, USA).

### 4.4. Strains and Culture Conditions

*Ustilaginoidea virens* strain P1 was cultured on PSA medium (containing 20% potato, 2% glucose, and 2% agar) at 28 °C. For RNA extraction, the strain was incubated on PSA plates covered with cellophane for two weeks.

The *A. oryzae* NSAR1 strain and its expression vectors (pTYGS-arg and pTYGS-met) used for heterologous expression were kindly provided by Professor Russell Cox at Leibniz University Hannover. Transformants and wild-type strains were cultivated on DPY medium (containing 2% dextrin, 1% polypeptone, 0.5% yeast extract, 0.5% potassium dihydrogen phosphate, and 0.5% magnesium sulfate hexahydrate, 1.5% agar) at 28 °C. For shake-culture experiments, strains were incubated in 100 mL of DPY medium at 28 °C with shaking at 180 rpm.

Plasmids used for yeast recombination and heterologous expression were constructed using *Saccharomyces cerevisiae* CEN.PK2. The strain was cultured on YPAD medium (containing 1% yeast extract, 2% tryptone, 2% glucose, 0.03% adenine, and 1.5% agar) at 30 °C. Correct transformants were selected on SM-URA selective medium (containing 0.67% YNB with ammonium sulfate, 2% glucose, 0.077% uracil-free complete supplement mixture, and 1.5% agar).

Cloning and plasmid amplification experiments were carried out using *Escherichia coli* DH5α. The strain was cultured on LA medium (containing 5% yeast extract, 10% tryptone, 10% sodium chloride, and 1.5% agar) at 37 °C. Corresponding antibiotics were added to the medium for transformant selection.

### 4.5. Bioinformatics Analysis

Genomic data were analyzed using the antiSMASH 7.0 software [19], and BLASTP 2.17.0+ program was employed to analyze gene functions (https://blast.ncbi.nlm.nih.gov/Blast.cgi, accessed on 24 May 2026). Multiple sequence alignment was performed using the online CLUSTALW tool (https://www.genome.jp/tools-bin/clustalw, accessed on 24 May 2026). Conserved domain prediction of proteins was carried out based on the Conserved Domain Database (CDD) of the National Center for Biotechnology Information (NCBI) [20].

### 4.6. UvSorB_PT Phylogenetic Tree Construction

The sequences of NR-PKSs with diverse cyclization modes were retrieved via literature mining ([3,21]) and NCBI BLASTP 2.17.0+ homology search using UvSorB as a query using DELTA-BLAST algorithm. For the purpose of retrieving PKSs with known products, the UniProtKB/Swiss-Prot database was searched, and only the sequences with identity >20%, query coverage >70% were used for analysis. Protein sequences were directly imported to MEGA 11, before sequence alignments using the CLUSTALW algorithm built within the software with default parameters. The phylogenetic tree was constructed via the neighbor-joining method using MEGA 11. A bootstrap test with 1000 replicates was conducted to assess phylogenetic confidence, and *Sus scrofa* FAS DH was designated as an outgroup. The final phylogenetic tree was visualized and optimized using the online platform iTOL (https://itol.embl.de/upload.cgi, accessed on 24 May 2026).

### 4.7. Construction of Heterologous Expression Vectors

For fragment-vector ligation based on yeast assembly technology, homologous sequences of more than 30 bp are required between the inserted fragments and the vector (Table S1). Two plasmids were selected according to different auxotrophic markers: pTYGS-arg for arginine auxotrophy and pTYGS-met for methionine auxotrophy. Both plasmids contain four conserved gene cloning sites, including P/TamyB, P/Tadh, P/TgpdA, and P/Teno, which allow insertion of target genes according to specific experimental requirements. Using pTYGS-UvSorB as the template, the upstream and downstream fragments of the PT and CMT domains were amplified separately. The two fragments were designed to possess homologous sequences with each other and homologous arms of no less than 30 bp to the vector. Yeast assembly was utilized to ligate the two fragments into the pTYGS-arg vector [13], and two knockout plasmids, pTYGS-arg-UvSorBΔPT and pTYGS-arg-UvSorBΔCMT, were finally constructed respectively (Figure S8).

### 4.8. Construction of Vector for Expressing Point-Mutated Gene

Using pTYGS-arg-UvSorB as the template, the UvSorB gene was divided into upstream and downstream segments and amplified separately. Single-point mutations were introduced at the 5′ end of the reverse primer for the upstream segment and the 3′ end of the forward primer for the downstream segment, respectively (Table S1). Subsequently, the upstream and downstream fragments with homologous arms were ligated into the pTYGS-arg vector via yeast homologous recombination [13]. Finally, four single-point mutation and one four-point mutations of the CMT domain were constructed, including pTYGS-arg-UvSorB-F1976M, -N1977L, -M2114F, -V2121F, and -Quadruple, respectively. Meanwhile, one single-point mutation and one double-point mutations of the PT domain were obtained, namely pTYGS-arg-UvSorB-Y1343H and -Y1343H/E1524D (Figure S9).

### 4.9. Transformation of A. oryzae NSAR1

The transformation of *A. oryzae* was performed according to the method described by Kahlert et al. [13]. Finally, PCR amplification was used to verify the identity of positive transformants (Table S1).

### 4.10. Fermentation and Extraction Procedures

Ten transformants were individually inoculated into 100 mL of DPY medium and incubated at 28 °C with shaking at 180 rpm for 7 days. Subsequently, the mycelium and culture filtrate were repeatedly extracted with ethyl acetate until the eluate became colorless. The ethyl acetate layer was dried over magnesium sulfate, filtered through filter paper, and concentrated under vacuum at 42 °C. The organic residue was redissolved in methanol and analyzed by HPLC and LC-MS. All experiments were performed in three independent biological replicates.

### 4.11. qRT-PCR Analysis

The strains were cultured on DPY plates covered with cellophane, and the cultures were continued until the mycelia fully covered the cellophane. Total RNA was extracted from the harvested mycelia using the Trizol method, and the purity and integrity of RNA were evaluated by spectrophotometry and agarose gel electrophoresis. High-quality RNA was reverse-transcribed into cDNA using a reverse transcription kit (Vazyme Biotech Co., Ltd., Nanjing, China).

qRT-PCR was performed in a total reaction volume of 10 μL with SYBR Green qPCR Master Mix (Vazyme) on a QuantStudio 7 real-time PCR system (Thermo Fisher Scientific, Waltham, MA, USA). The primers used in qRT-PCR were listed in Table S2. The amplification program was set as follows: initial denaturation at 95 °C for 60 s, followed by 40 cycles of 95 °C for 3 s, 54 °C for 30 s and 72 °C for 10 s. Subsequently, the samples were incubated at 95 °C for 15 s and 60 °C for 60 s. Melting curve analysis was conducted by gradually increasing the temperature from 60 °C to 95 °C to confirm the specificity of amplified products. Three independent transformants of each mutant were selected as biological replicates, and each qRT-PCR detection was performed with three technical replicates. The relative gene expression levels were calculated using the 2^−Δ*Ct*^ method with a reference gene (actin) for normalization.

### 4.12. Statistical Analysis

All data were statistically analyzed using SPSS 27.0 software. One-way analysis of variance (ANOVA) was used for inter-group difference comparison, and LSD method and Duncan’s multiple range test was applied for post hoc multiple comparisons. A *p* value less than 0.05 (*p* < 0.05) was defined as the threshold for statistically significant differences. All experimental results were presented as bar graphs with error bars and significance markers.

## 5. Conclusions

Fungal NR-PKSs with an apparently inactive PT domain constitute a small group of NR-PKS, which make use of Knoevenagel reaction and Claisen condensation to construct an aromatic ring. Using the sorbicillin NR-PKS SorB as an example, we found out that removal of the PT domain did not change the polyketide profile, but decreased yield. This was consistent with the lower transcriptional level of the knockout mutant. Phylogenetic analysis suggested that the mutation at key catalytic dyad residues might render the PT nonfunctional. However, reverting these residues with the conserved residues did not lead to any products corresponding to a functional PT. Meanwhile, removal of the CMT stopped the polyketide production, highlighting its important role in the in vivo polyketide biosynthesis. The methylation pattern was correlated with the substrate-binding residues of the CMT domain, regardless of the starter unit. Site-directed mutagenesis to CMT of *UvSorB* was carried out in an attempt to alter the methylation pattern. These led to abolishment of any sorbicillin-type polyketides. The transcriptional analysis revealed a significantly lower level of these CMT mutants compared to the wild type. These results have deepened our understanding of the programming of fungal NR-PKS.

## Data Availability

The data supporting this article have been included in the Supporting Information.

## Supplementary Materials

The following supporting information can be downloaded at: https://www.mdpi.com/article/10.3390/ijms27125534/s1 [10,13,22].
