# Supplementary material for: Engineering a Fungal Non-Reducing Polyketide Synthase with an Apparently Inactive Product-Template Domain Reveals Insights into the Catalytic Reprogramming
Source: Int J Mol Sci. 2026 Jun 18;27(12):5534. doi: 10.3390/ijms27125534 (PMC13300579; doi:10.3390/ijms27125534)
Supplement: Supplementary file 1 [file ijms-27-05534-s001.zip › ijms-4332890-supplementary.pdf]

Table S1. Primers used in this study

Primer name	Sequence (5' to 3')	Description
SorBSAT-F+PamyB	ctgaacaataaaccccacagcaagctccgaATGGCGCGCACGGCGACGCA	To construct the pTYGS-arg-UvSorB ΔCMT heterologous expression vector
SorBKOCMeT-1R	GTCGCAGCTGTACTTGGCT	
SorBKOCMeT-1R PLUS	TCCAGGGGGCAAACCATGGTGGCTGTCGCCGTCGCAGCTGTACTTGGCTGAGTA	
SorBKOCMeT-2F	ACAGCCACCATGGTTTGC	
SorBKOCMeT-2F PLUS	ATAGAATACTCAGCCAAGTACAGCTGCGACGGCGACAGCCACCATGGTTTGC	
SorB-R+TamyB	catatactctccacccttcacgagctactacagatCTACCGCAAAAATCCAGC	
SorBKOPT-1R	TTCTCGGCGATTCATCAGCT	To construct the pTYGS-arg-UvSorB ΔPT heterologous expression vector
SorBKOPT-1R PLUS	CACGAAACTCGAACCAGCGCGCACCTCAACTTCTCGGCGATTCATCAGCT	
SorBKOPT-2F	TGGTTCGAGTTTCGTGCA	
SorBKOPT-2F PLUS	CCCAGCTGATGAATCGCCGAGAAGTTGAGGTGCGCGCTGGTTCGAGTTTCGTGCA	
SorB-F-FMNL	GGCGAGCTTCCCATGTTGAAGTTGTGCTTT	To construct the heterologous expression vector of pTYGS-arg-UvSorB with CMT domain mutations
SorB-R-FMNL	AAAGCACAACTTCAACATGGGAAGCTCGCC	
SorB-F-MFVF	ATGGGAACCTTGCATTGGTTTGATGTGGTT	
SorB-R-MFVF	ATGCAAGGTTCCCATAAACTCCAGCATCAT	
SorB-F+YH	AAGTCGAATCGCAGCATTTGCTGGCCAATT	To construct the heterologous expression vector of pTYGS-arg-UvSorB with CMT domain mutations
SorB-R+YH	AATTGGCCAGCAAATGCTGCGATTCGACTT	
SorB-F+ED	TTTGCACTCGGTGATAGCTTCACGCAAGTG	
SorB-R+ED	CACTTGCGTGAAGCTATCACCGAGTGCAAA	
CMeT CHECK-F	ACTCGAATCGTCAACATCGA	To verify the inserted fragments
CMeT CHECK-R	TTCAGAACCGTGGCTTCT	
PTCHECK-F	TCCATGACTGACTCGGCT	
PTCHECK-R	GCGTCAAAGACAAAGATGT	

Table S2. Primers used in qRT-PCR analysis

Primer name	Sequence (5' to 3')	Description
AO actin-FL(qRT)	ACGCCCCCGCCTTCTACGTCTCCATCCAGG	To determine the expression level of the reference gene
AO actin-RL(qRT)	CGGCCAGCCATGTCAACACGGGAGATGGCG	
6010KS-F1(qRT)	AATACTGGGACTTACTGCTCGAAGGCCGGT	To determine the expression level of the *UvSorB-KS domain*
6010KS-R1(qRT)	CCTGGTACGCAGTCTGCAAAATCAGCCTCT	
6010KS-F3(qRT)	ATTCGACAAGTGTTTGGCGGTCCTGAACGC	
6010KS-R3(qRT)	GCTGCCTTCCCAAGGCAGAGAAACCTTTGT	
6010CMT-F1(qRT)	AACGCTCAGGATTTTGGAAATGGGAGCTGG	To determine the expression level of the *UvSorB-CMT domain*
6010CMT-R1(qRT)	TGCTGAGAACCTATCAAGTCGGCATCGCCC	
6010CMT-F2(qRT)	TGCACAAAACATCCGCAAGTTCCTGCGTCC	
6010CMT-R2(qRT)	CTTGCCGTCCGTCCATTCAACGTGCTTGTA	

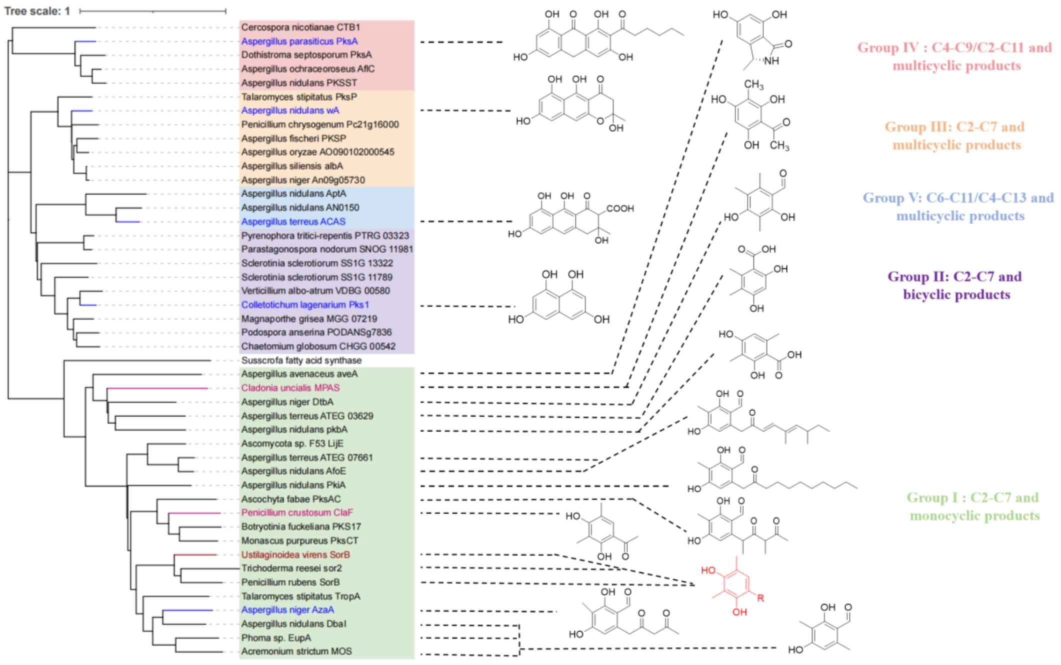

Figure S1. Phylogenetic analysis of the UvSorB PT domain with group I-V PT domains (full tree). *Sus scrofa* FAS DH domain was selected as an outgroup. The UvSorB PT domain is indicated with a red line. The representative PT in each group was indicated with a blue line, with its product shown nearby. The ClaF_PT and MPAS_PT are shown in purple, which seems non-functional judging from the structure of clavatol and 3-methylphloracetophenone.

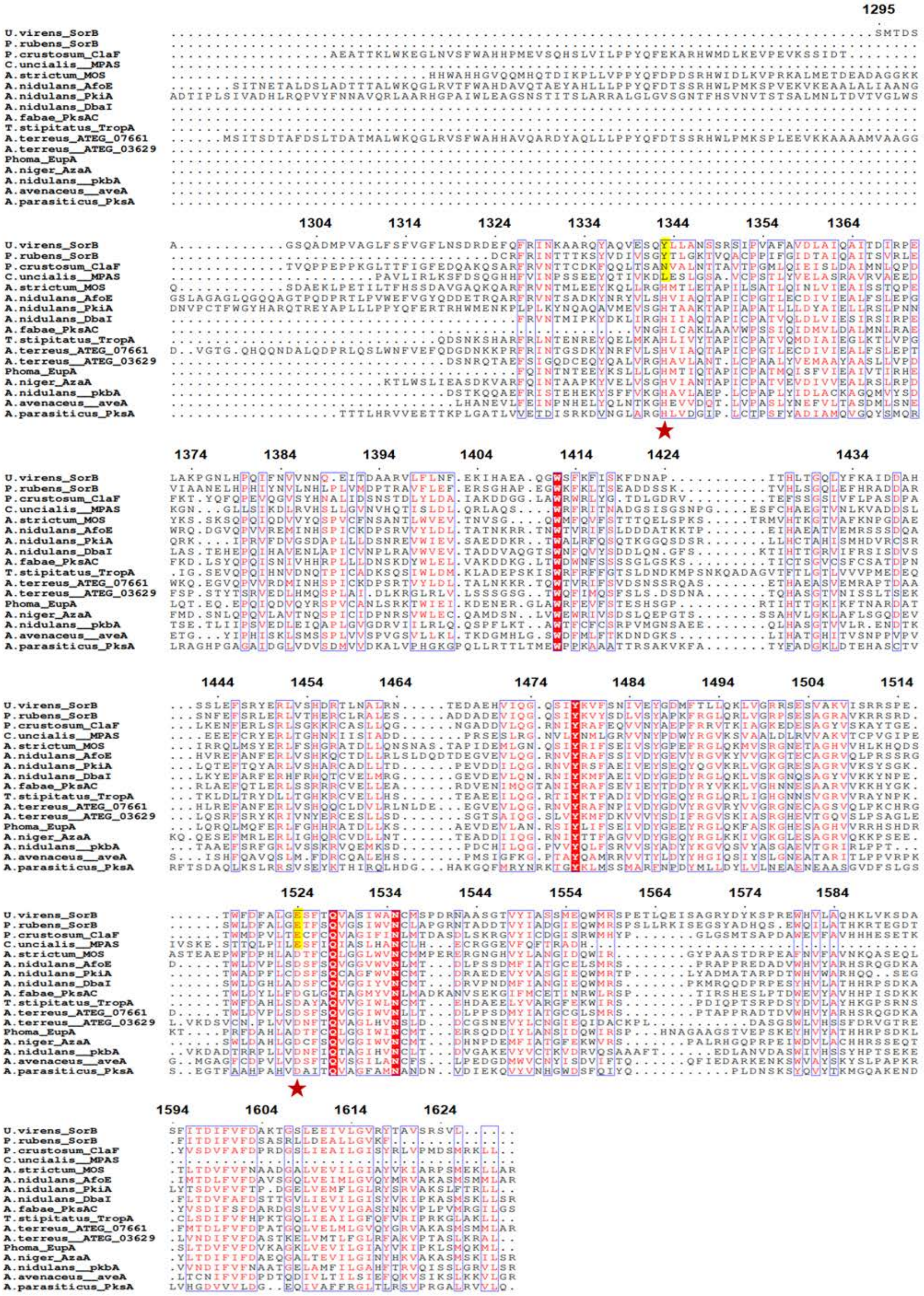

Figure S2. Multiple sequence alignment of the *UvSorB*_PT domain with group I PT domains (except PksA). The sequences were obtained from NCBI.

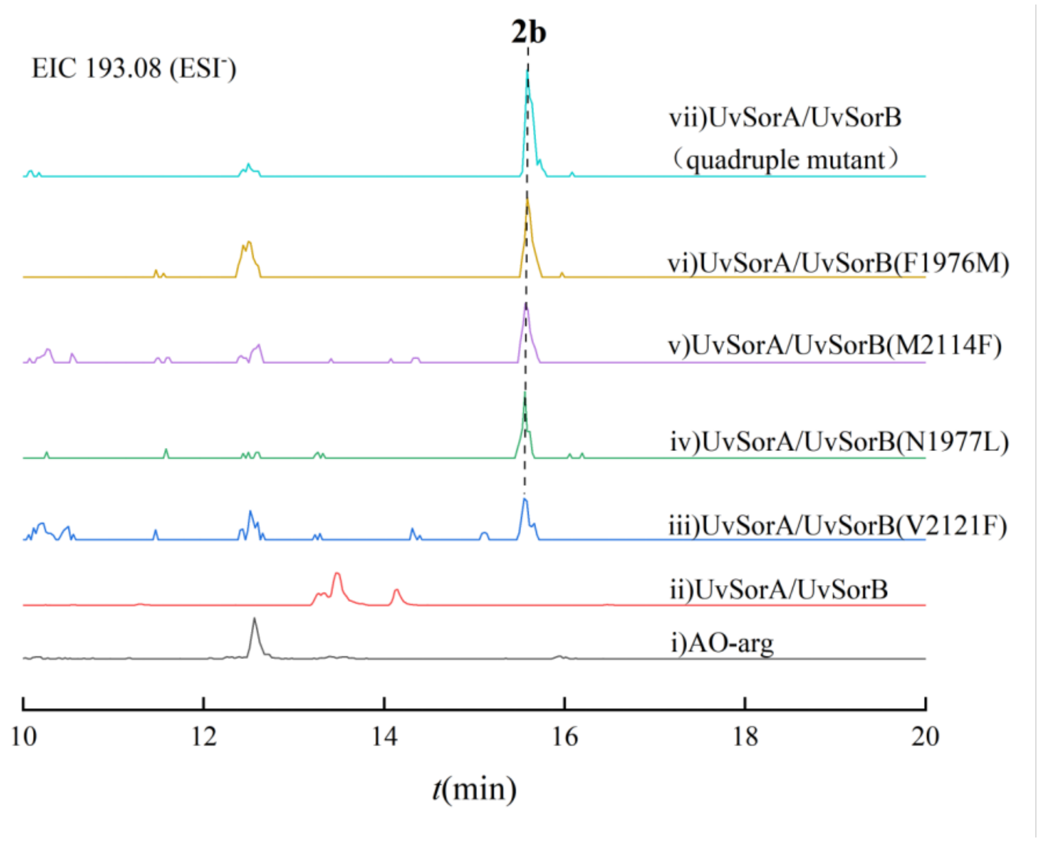

Figure S3. EIC spectra (*m/z* 193.08) of the extract from co-expression of *UvSorA* and various CMT mutants of *UvSorB* in *A. oryzae* (LC-MS, negative mode). i) empty vector control; ii) *UvSorB*; iii–vi) single mutation, and vii) quadruple mutations of *UvSorB*.

Compound characterization

sorbicillin (**1a**):

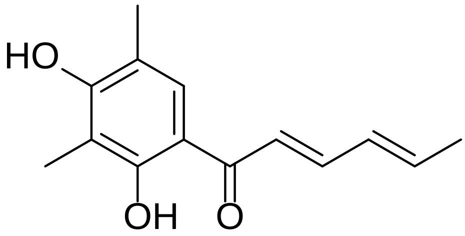

Chemical Formula: $C_{14}H_{16}O_3$; Exact Mass: 232.11
UV λ_{max}(CH_3CN): 200, 230, 322 nm
HRESIMS: *m/z* $[M+H]^+$ calcd for $C_{14}H_{17}O_3$: 233.1172, found: 233.1174
Compound was identified based on mass, retention time and UV-absorption with the standard [10].

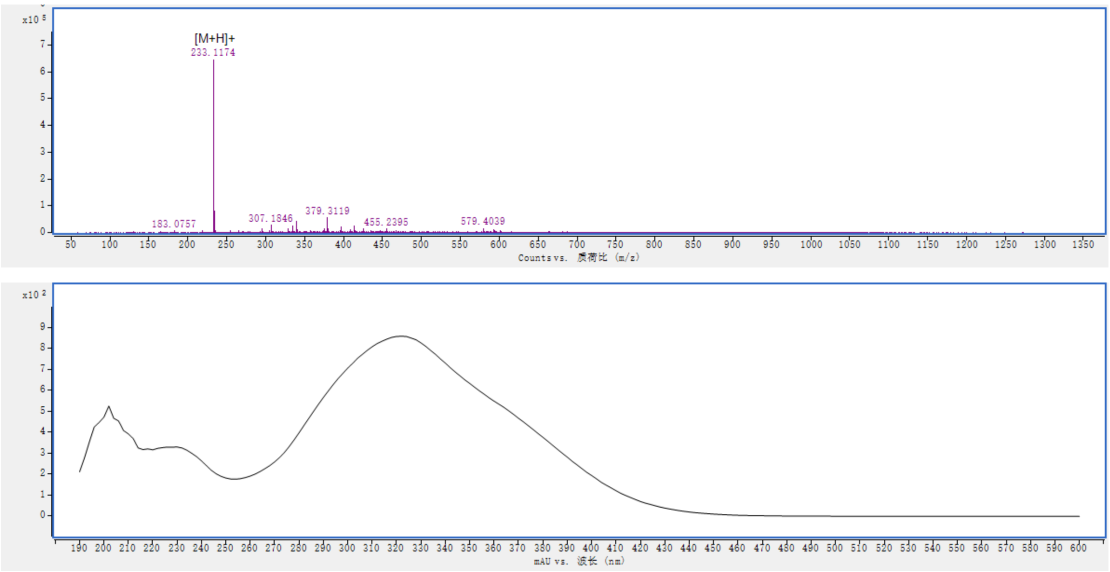

Figure S4. HRESIMS (top) and UV (bottom) spectra of **1a**.

2',3'-dihydrosorbicillin (**1b**):

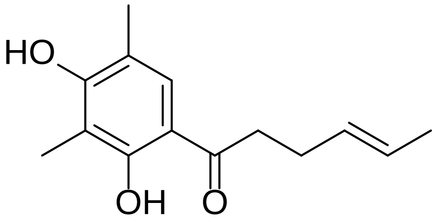

Chemical Formula: $C_{14}H_{18}O_3$; Exact Mass: 234.13

UV λ_{max}(CH_3CN): 220, 283, 332 nm

HRESIMS: *m/z* $[M+H]^+$ calcd for $C_{14}H_{17}O_3$: 235.1329, found: 235.1324

Compound was identified based on high-resolution mass, and UV-absorption analysis by comparing to the literature where the same compound was found in co-expression of SorA/SorB in *Aspergillus oryzae* NSAR1 [13].

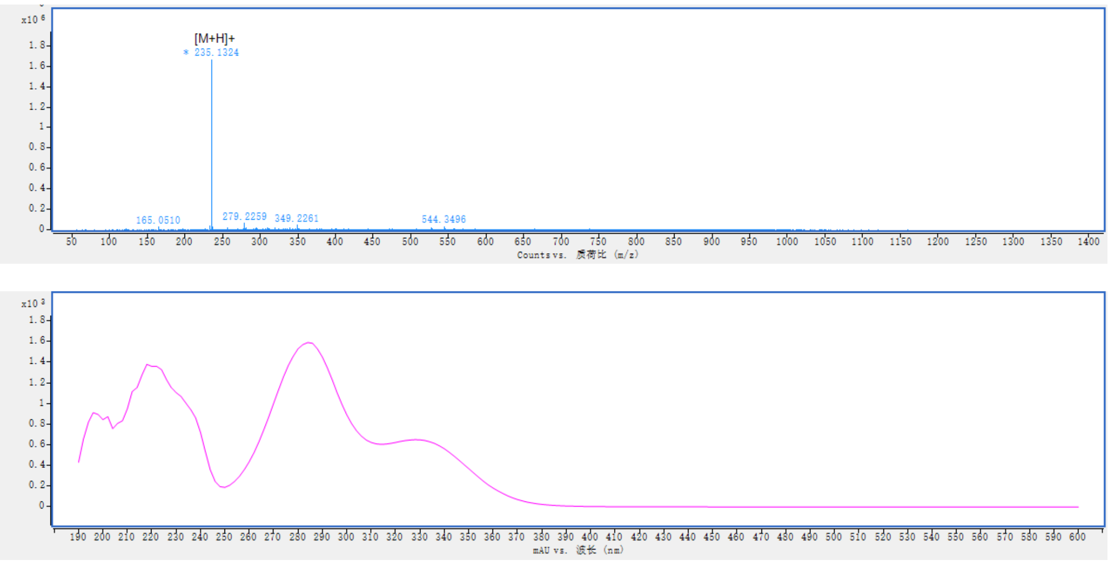

Figure S5. HRESIMS (top) and UV (bottom) spectra of **1b**.

trichopyrone (**2a**):

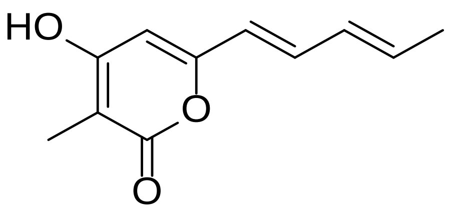

Chemical Formula: $C_{11}H_{12}O_3$; Exact Mass: 192.08

UV λ_{max}(CH_3CN): 245, 345 nm

HRESIMS: *m/z* $[M-H]^-$ calcd for $C_{14}H_{17}O_3$: 191.0714, found: 191.0705

Compound was identified based on high-resolution mass, and UV-absorption data by comparing with the literature [22].

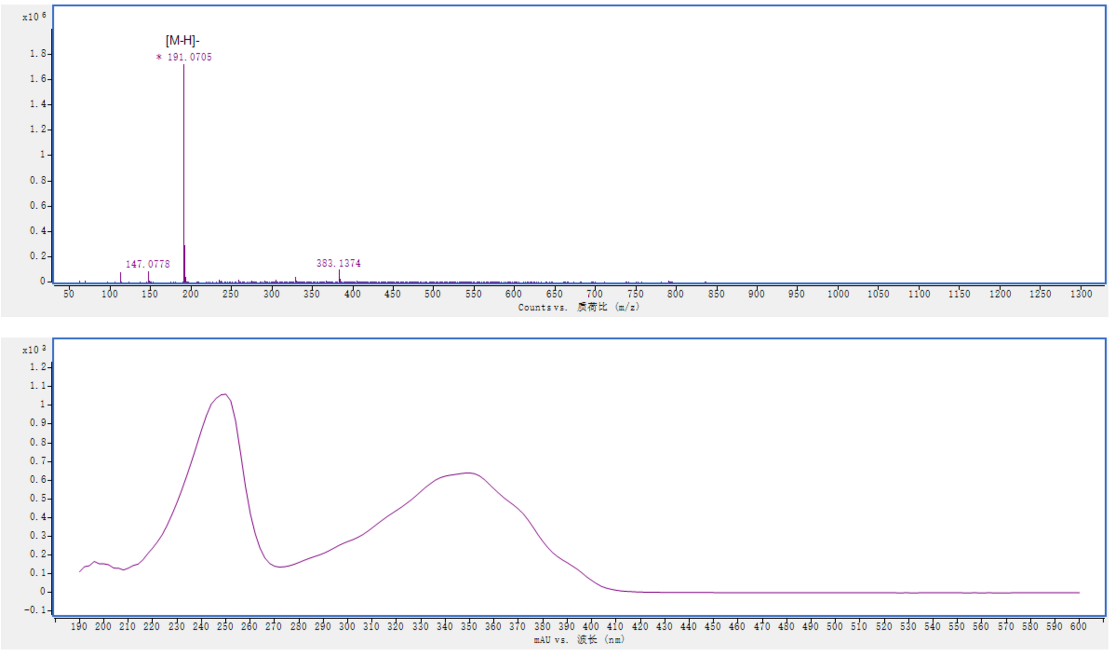

Figure S6. HRESIMS (top) and UV (bottom) spectra of **2a**.

2b: structure unknown

Chemical Formula: $C_{11}H_{14}O_3$; Exact Mass: 194.09

UV λ_{max}(CH_3CN): 222, 280 nm

HRESIMS: *m/z* [M-H]$^-$ calcd for $C_{11}H_{13}O_3$: 193.0870, found: 193.0854

For HRESIMS, MS/MS spectra, and UV spectra see figure S7 .

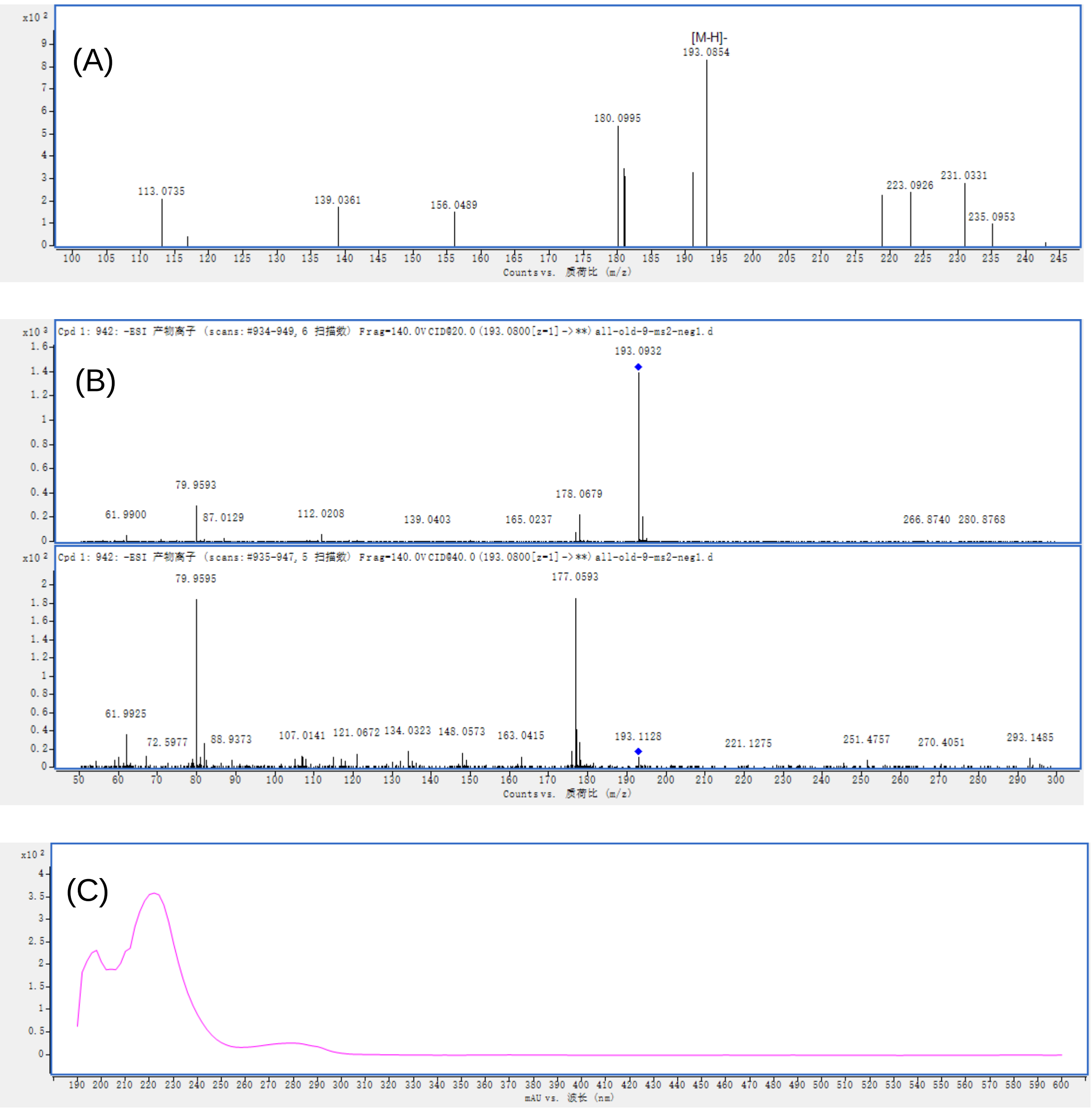

Figure S7. HRESIMS (A), MS/MS (B), and UV (C) spectra of **2b**.

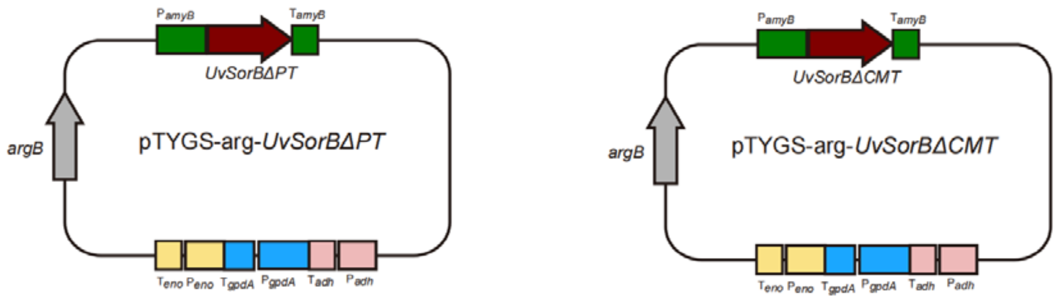

Figure S8. Plasmids for heterologous expression of UvSorB without the PT and CMT domains in *A. oryzae.*

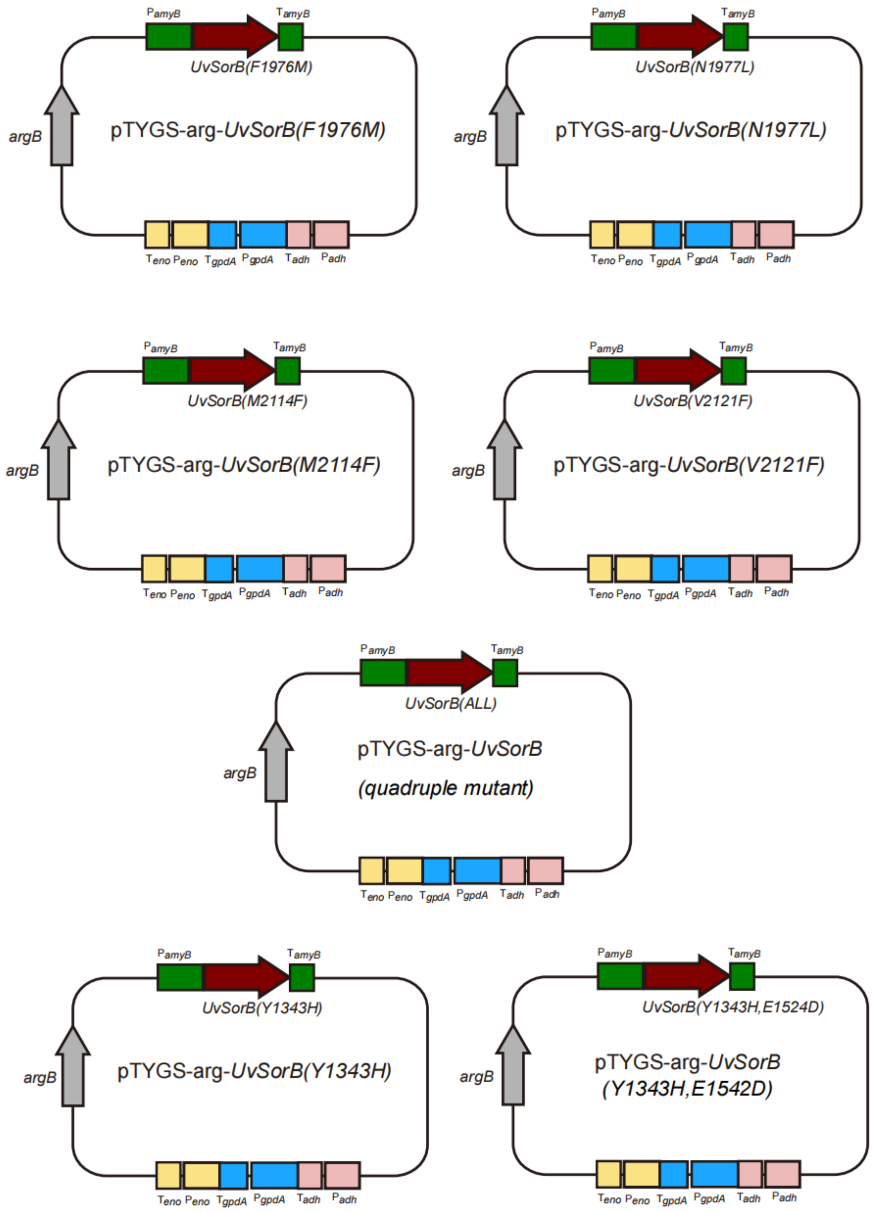

Figure S9. Plasmids for heterologous expression of mutated PT and CMT (point mutation were indicated) in *A. oryzae.*
